# HER2 expression as a potential marker for response to therapy targeted to the EGFR

**DOI:** 10.1038/sj.bjc.6603078

**Published:** 2006-04-04

**Authors:** D R Emlet, R Schwartz, K A Brown, A A Pollice, C A Smith, S E Shackney

**Affiliations:** 1 Laboratory of Cancer Cell Biology and Genetics, Department of Human Oncology, Drexel University College of Medicine, Allegheny General Hospital, 320 East North Avenue, Pittsburgh, PA 15212, USA; 2 Department of Biological Sciences, Carnegie Mellon University, Pittsburgh, PA 15213, USA

**Keywords:** HER2, EGFR, HER3, trastuzumab, targeted therapy, breast cancer

## Abstract

Since human epidermal growth factor receptor 2 (HER2) is known to participate with the epidermal growth factor receptor (EGFR) in mitogenic signalling, we hypothesised that HER2 overexpression might indicate responsiveness to EGFR targeted therapies. MCF7 breast cancer cells transfected with the HER2 gene were subcloned to establish a set of genetically related cell lines expressing graded levels of HER2 by immunoblot analysis. The subcloned cell lines and parental MCF7 cells were characterised by their growth characteristics, and cell by cell patterns of EGFR, HER2 and HER3 expression as well as levels of phosphorylated mitogen-activated protein kinase (MAPK) and AKT by laser scanning cytometry (LSC). Growth inhibition assays were used to characterise response to EGFR targeted therapy, and to determine the relationship between therapeutic response and levels of tyrosine kinase expression. The levels of growth inhibition of AG1478 and of the AG1478-trastuzumab combinations were correlated with levels of HER2 expression among the different cell lines. Among EGFR, HER2 and HER3, HER2 overexpression was the best single predictive marker, but combinations of two markers provided additional predictive information.

Human epidermal growth factor receptor 2 (HER2), a member of the epidermal growth factor receptor (EGFR) family of tyrosine kinases, is overexpressed in 25–30% of human breast cancers (Slamon *et al*, 1987, 1989). It has been implicated in cancer progression (Yarden, 2001; Nahta and Esteva, 2003), and has been identified as a prognostic and predictive marker for breast cancer outcome (Winston *et al*, 2004). Breast cancers with HER2 gene amplification and/or protein overexpression have been shown to be responsive to trastuzumab (Herceptin), a humanised monoclonal antibody directed against the extracellular domain of HER2 (Baselga, 2001; Arteaga and Baselga, 2004). Trastuzumab produces high objective response rates and prolongation of overall survival as a single agent (Vogel *et al*, 2002) and in combination with chemotherapy (Slamon *et al*, 2001). Several assays, based on immunohistochemistry (IHC) or fluorescence *in situ* hybridisation (FISH), are FDA-approved for the clinical identification of breast cancer patients who are likely to respond to trastuzumab (Winston *et al*, 2004). The clinical usefulness of trastuzumab in breast cancer may be due in part to the robustness and wide availability of these standardised assays.

The EGFR is expressed in 15–35% of breast cancers, and is also associated with a poor prognosis (Thor *et al*, 2001; Suo *et al*, 2002). Agents targeted against the EGFR have been studied extensively in the laboratory, and several have undergone clinical trials, including Cetuximab (Erbitux), a humanised monoclonal antibody directed against the extracellular domain of the EGFR, and the small molecule tyrosine kinase inhibitors (TKIs) Gefitinib (Iressa/ZD1839), and Erlotinib (Tarceva/OSI-774). These agents have demonstrated clinical activity in 8–20% of patients with non-small-cell lung cancer (NSCLC), especially in a subset of NSCLC patients whose tumours contain mutations involving the ATP binding pocket of the EGFR (Lynch *et al*, 2004; Paez *et al*, 2004). Although preclinical cell culture and xenograft studies suggest that EGFR targeted therapies hold promise for certain subtypes of breast cancer, clinical trials to date have shown responses in less than 10% of patients (Konecny *et al*, 2003; Arteaga and Truica, 2004; Kaklamani and O'Regan, 2004).

Tumour EGFR expression has not proven to be a useful predictive marker of clinical response to EGFR-targeted therapies (Johnson and Arteaga, 2003; Campiglio *et al*, 2004). There is no clear correlation between EGFR expression and response to Erlotinib, Gefitinib or Cetuximab efficacy in NSCLC or colorectal cancer (Saltz *et al*, 2001; Fukuoka *et al*, 2003; Johnson and Arteaga, 2003; Perez-Soler, 2004; Saltz *et al*, 2004; Noberasco *et al*, 2005). This may be due in part to the lack of a standardised protocol and grading system for EGFR expression in clinical samples (Noberasco *et al*, 2005; Younes, 2005), to technical limitations that are inherent in immunohistochemical methods (Younes, 2005), or, perhaps, to an intrinsically poor correlation between the level of EGFR expression and therapeutic response (Arteaga and Baselga, 2004).

Robust predictive markers are needed in order to identify the relatively small subsets of patients whose tumours are likely to respond to EGFR-targeted therapies (Baselga, 2002; Castro, 2002; Arteaga and Baselga, 2003). Candidate markers include phosphorylated EGFR (Arteaga and Baselga, 2003; DiGiovanna *et al*, 2005), and phosphorylated effector molecules downstream of the EGFR, such as the mitogen-activated protein kinase (MAPK) and protein kinase B (AKT) (Lynch *et al*, 2004; Han *et al*, 2005). However, there are concerns about the stability of phosphorylated proteins in primary tumour samples prior to fixation, and protocols for the collection and processing of clinical material for phosphorylated protein analysis have yet to be validated and standardised (Baselga, 2002; Lynch *et al*, 2004).

Human epidermal growth factor receptor 2 and the EGFR are known to heterodimerise with one another to drive mitogenic signalling pathways, and heterodimerisation has been shown to inhibit internalisation and degradation of the EGFR (Lenferink *et al*, 1998). Thus, receptor interactions involving HER2 may play key roles in breast cancer cells that both overexpress HER2 and express the EGFR. Epidermal growth factor receptor inhibitors are effective in blocking HER2 mediated signalling in HER2 overexpressing breast cancer cell lines that co-express the EGFR, and combinations of EGFR and HER2 targeted agents have been shown to have additive or synergistic effects on growth inhibition (Moasser *et al*, 2001; Moulder *et al*, 2001; Normanno *et al*, 2002; Anido *et al*, 2003). A recent clinical study has suggested that the adverse prognostic value of HER2 overexpression occurs only with HER2 activation or EGFR co-expression in breast cancer (DiGiovanna *et al*, 2005). In another recent clinical study in NSCLC, increased HER2 gene copy number was associated with sensitivity to gefitinib in EGFR-positive patients (Cappuzzo *et al*, 2005).

To investigate the potential role of HER2 as a predictive marker for responsiveness to EGFR targeted therapies, we developed a series of MCF7 breast cancer cell line subclones that express graded levels of HER2. Despite heterogeneity of levels of EGFR and HER3 expression, levels of MAPK and AKT phosphorylation, and overall growth rates among the different cell lines, the inhibition of tumour cell proliferation by AG1478 alone or in combination with trastuzumab was correlated with levels of expression of HER2.

## MATERIALS AND METHODS

### Generation of HER2 transfectant cell lines

cDNA encoding human HER2 was excised from a pLXSN retroviral expression construct (Riese *et al*, 1995) by digestion with *Xho*1, and inserted into the mammalian expression vector pcDNA 3.1 encoding geneticin resistance. Plasmids containing the correctly oriented insert were identified by restriction digest mapping followed by sequencing of the 5′ and 3′ ends of the insert. The HER2-pcDNA 3.1 construct was transfected into the breast cancer cell line MCF7 utilising a BioRad Gene Pulser Xcell. MCF7 cells were grown to 80–90% confluence and fed the day before transfection. Cells were trypsinised, centrifuged, and resuspended at a concentration of 1 × 10^7^ cells ml^−1^ in culture medium. A measure of 400 *μ*l of the cell suspension was mixed with 100 *μ*g of DNA construct in a 0.4 cm gap electroporation cuvette (BioRad, Hercules, CA, USA), and incubated at 4°C for 10 min on ice. The transfection mixture was electroporated at 220 V and 950 *μ*F and plated into two 100 mm tissue culture plates. The transfected cells were cultured overnight for recovery and then cultured in the appropriate selective media for 4–8 weeks. Resultant colonies or cell pools were subcloned by colony transfer and plating of cell pools into 24-well plates at an average ratio of 2 cells per well. Individual neomycin resistant clones were expanded and assayed for HER2 expression by immunoblot analysis. SKBR3 (an unrelated HER2 overexpressing breast cancer cell line) was used as an independent comparative reference for HER2 expression in the immunoblots.

### Cell culture and preparation

JC 1939, MCF7, and SKBR3 breast cancer cell lines were maintained in RPMI 1640 containing 100 U ml^−1^ penicillin and streptomycin, 0.25 *μ*g ml^−1^. amphotericin B, and 10% fetal bovine serum (FBS). The HER2 transfected clonal cell lines were cultured in the same media supplemented with 500 *μ*g ml^−1^ geneticin.

### Immunoblot analysis

Cell lysate (50 *μ*g) was subject to SDS–PAGE on 4–20% tris/glycine gels, transferred onto nitrocellulose filters, and blocked for 0.5 h in TTBS (100 mM Tris, pH 7.5, 0.9% NaCl, 0.1% Tween-20) with 5% nonfat dry milk. A monoclonal antibody against HER2 (clone CB11, Novacastra Laboratories, Newcastle-upon-Tyne, UK) was used at a 1 : 1000 dilution. The secondary antibody was goat anti-mouse horseradish peroxidase (HRP) conjugated antibody (Santa Cruz Biotechnology), and was used at a 1 : 1000 dilution. Antibody signal was visualised using the SuperSignal West Pico Chemiluminescent Substrate (Pierce) exposed to Kodak BioMax XAR film. Densitometry was performed using a Personal Densitometer SI (Molecular Dynamics), and the data were analyzed using Scion Image for Windows (Scion Corporation, Medford, MA, USA).

### Laser scanning cytometry (LSC) analysis

For LSC analysis, cell lines plated into 75 cm^2^ flasks at 2.5 × 10^6^ cells flask^−1^ and harvested in log phase growth by trypsinisation were treated with cold (4°C) 5 mM dithiothreitol (DTT) for 15 min at room temperature to reduce clumping and fixed in 0.5% paraformaldehyde and 70% methanol as described previously (Pollice *et al*, 1992). Aliquots of 2 × 10^4^ cells from each of the cell lines were filtered through 64 *μ*m nylon mesh (Small Parts), and centrifuged at 200 **g** for 2 min. For EGFR family member analysis, monoclonal antibodies against HER2 (clone CB11, Novacastra Laboratories), EGFR (clone EGFR.1, BD Pharmingen, San Diego, CA, USA), and HER3 (clone Ab 5, Oncogene Research Products, La Jolla, CA, USA), directly conjugated to FITC, PE and Cy5, respectively, were used at a 1 : 10 dilution in a 1-h incubation at room temperature in the dark. For the phosphorylated (P) MAPK and AKT analysis, monoclonal antibodies against P-MAPK (clone E10, Cell Signaling Technology, Beverly, MA, USA) and P-AKT (clone 4E2, Cell Signaling Technology) directly conjugated to Cy5 and Cy3, respectively, were used. Cell suspensions were washed 1 × with PBS and resuspended in 100 *μ*l of 4′,6-diamidino-2-phenylindole (DAPI) (Sigma) at a final concentration of 1 *μ*g ml^−1^ in v v^−1^ 1 : 1 glycerol : PBS. 100 *μ*l of cell suspension was pipetted into a HybriWell chamber (22 × 22 × 0.15 mm^3^) (Schleicher&Schuell) affixed to precleaned glass microscope slides. Fluorescence measurements were made using a laser scanning cytometer (LSC) (CompuCyte) with the WinCyte (version 3.6) program, equipped with an air-cooled violet diode laser emitting at a wavelength of 405 nm, an air-cooled argon laser emitting at a wavelength of 488 nm, and an a HeNe laser emitting at a wavelength of 633 nm. DAPI fluorescence was measured using a 463/39 nm band pass filter, FITC fluorescence was measured using a 530/30 nm band pass filter with a 555-nm dichroic long pass filter, CY3 fluorescence was measured using a 580/30-nm band pass filter with 605-nm dichroic long pass filter and CY5 fluorescence was measured using a 650-nm long pass filter with a full mirror. A fixed scan area of 1.6 × 10^8^ *μ*m^2^ centred on the HybriWell chamber was used with all samples. DNA was used as the contouring parameter with a threshold of 700 and 30 pixels added to threshold. For quantitation of EGFR, HER2 and HER3 in molecules per cell, the reference cell line JC 1939 was subjected to ELISAs (Oncogene Science, Carpenteria, CA, USA) with the appropriate antibodies. Fluorescence signal in each cell line from LSC analysis was normalised to the signal from JC 1939 to determine molecules per cell.

### Proliferation assays/inhibitor analysis

The EGFR inhibitor AG1478 was purchased from Calbiochem, La Jolla, CA, USA, and was stored at −20°C as a 10 mM stock in DMSO. Trastuzumab was a gift from Genentech. Cell stocks were grown to 80% confluence, trypsinised, and plated in triplicate into 12-well tissue culture plates at 2 × 10^4^ cells well^−1^. The following day, duplicate wells (for day 0 counts) were trypsinised and each well was counted in triplicate with a haemocytometer. The remaining wells were fed every other day with regular cell culture media supplemented with appropriate inhibitor or DMSO as vehicle control. On day 6, the remaining wells were trypsinised and counted in triplicate with a hemocytometer, and the data were graphed in Excel.

### Statistical analysis

Statistical analyses were performed using SPSS version 12.0 for Windows. Human epidermal growth factor receptor 2 expression, cell growth and drug treatment data were analyzed by one-way ANOVA followed by a two-sided Dunnett's test *post hoc* for the determination of differences between groups. Pearson correlation coefficients of linear regression were calculated for −log of surviving cell fraction *vs* relative HER2 expression.

The combination index (CI) for drug effect in each cell line was calculated from the surviving cell fractions of cells treated with each drug alone, SF_A_ and SF_B_, and the surviving cell fractions of cells treated with the drug combination, SF_AB_, where CI=SF_AB_/(SF_A_ × SF_B_). Triplicate analyses were subjected to two-sided statistical tests (1-group, two-tailed test with degrees of freedom=2) to determine if the mean CI value for each cell line was significantly different from a CI of 1.0 at the *P*<0.05 level. Synergy was defined by a CI value that was significantly >1.0, and antagonism was defined by a CI value significantly greater than 1.0. Effects were considered additive if the CI was not significantly different from 1.0.

### Prediction of treatment outcomes

Least-squares linear regression analysis was applied to correlate protein expression levels with efficacy ratios of trastuzumab and AG1478 on the parental MCF7 and HER2 expressing subclones. Mean expression of EGFR, HER2 and HER3 were derived from cell-based measurements of molecules per cell by LSC. Drug efficacy ratios were derived for each treatment by taking one minus the ratio of cells counted following treatment to cells counted without treatment. A linear regression model was defined to relate efficacy ratio (*k*) to mean expression levels of EGFR, HER2 and HER3 using the formula 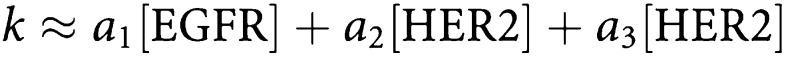 Constants *a*_1_, *a*_2_ and *a*_3_ were derived to provide a least-squares fit between observed and predicted efficacy ratios across the cell lines examined. Least-squares regression was further attempted for each of the three assayed genes in isolation, using models of the form 
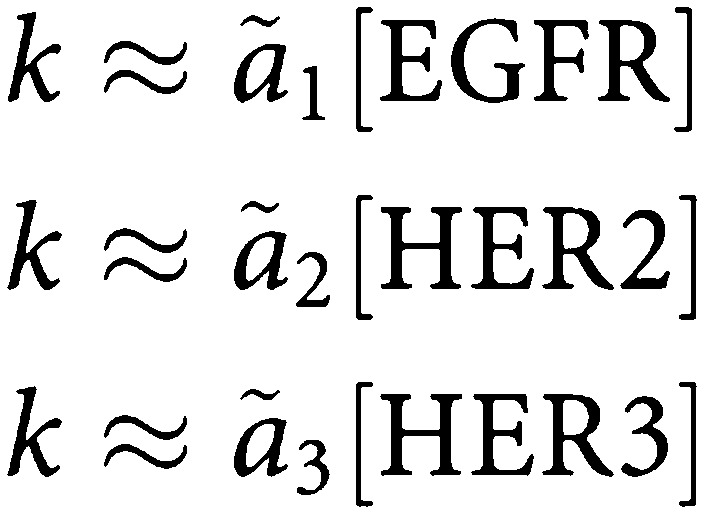
 where each of the *ã*_1_, *ã*_2_ and *ã*_3_ constants was derived to provide a least-squares best-fit between the observed efficacy ratios and those predicted from the single gene in isolation. Each of these linear regression analyses was tested by leave-one-out cross validation. For each cell line, parameters were fit without using the data provided by that cell line and the efficacy ratio for the missing cell line was predicted from the derived parameters. The root mean square errors of the predictions were assessed for all regression analyses with and without the cross-validation.

## RESULTS

### Generation of MCF7 subclones that overexpress HER2 at graded levels, and analysis of growth characteristics

**Figure 1:**
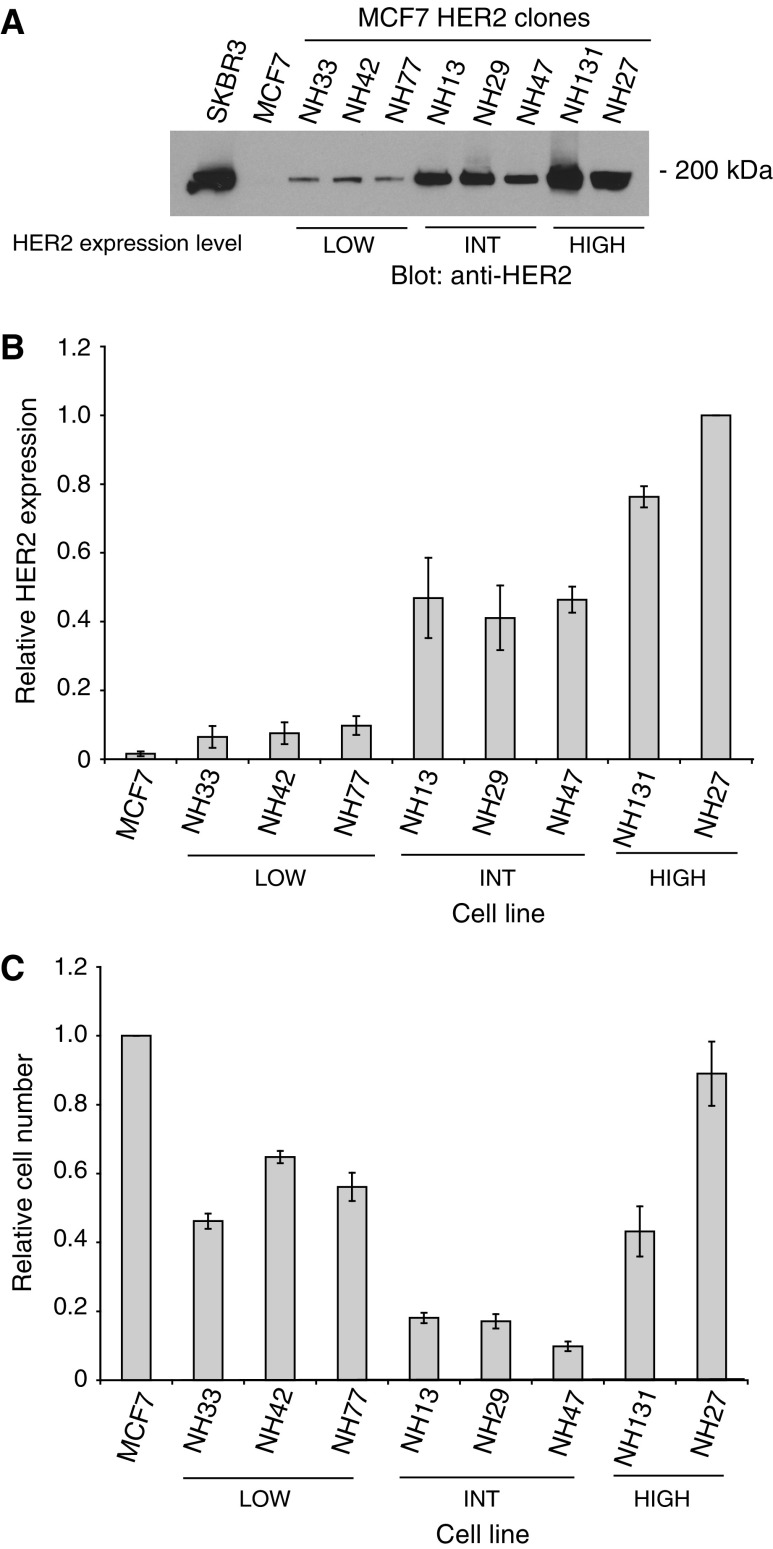
Stable HER2 expression levels and growth characteristics of HER2 transfected MCF7 subclones. (**A**) HER2 expression by immunoblot analysis in HER2 cDNA vector transfected MCF7 cell lines overexpressing low (LOW), intermediate (INT) and high (HIGH) levels of HER2 compared to parental MCF7 and SKBR3 breast cancer cell lines. The position of the molecular weight marker is shown on the right. (**B**) Exposures from three separate immunoblots of the MCF7 subclones were subjected to densitometry, and density values were normalised to the HER2 value from the NH27 cell line. The means of the HER2 signals from the LOW, INT and HIGH groups are statistically significantly different from one another and from the MCF7 signal (*P*<0.01 in each comparison), except for the MCF7 *vs* the LOW HER2 expressing subclones (*P*=0.462). (**C**) Total cell number relative to control MCF7 after 6 days of growth for the HER2 transfected MCF7 subclones. Results of three independent experiments were normalised to the mean of parental MCF7 cells. Bars, ±s.e.

The MCF7 breast cancer cell line was transfected with a pcDNA 3.1 construct encoding human HER2 that was derived from a retroviral construct (Riese *et al*, 1995), and HER2 expressing clones were identified by immunoblot analysis (Figure 1A). Clones were isolated that overexpress low (NH33, NH42 and NH77), intermediate (NH13, NH29 and NH47), or high (NH131 and NH27) levels of HER2 by comparison with the parental MCF7 line. The cell lines with the highest level of HER2 expression exhibit levels that approach that found in the endogenously high HER2 overexpressing SKBR3 cell line. Quantitative densitometry measurements on HER2 immunoblots from multiple passages of these clones are shown in Figure 1B. The growth rates and levels of HER2 expression of the subclones were monitored at each passage to ensure stability.

The relative growth rates of the transfected cell lines are shown in Figure 1C. The HER2 transfected clones all grow more slowly than the reference parental MCF7 line. This is consistent with previous studies that have demonstrated a decrease in the growth rate of MCF7 cells with HER2 transfection and overexpression (Giani *et al*, 1998; Huang *et al*, 2002). The low HER2 overexpressing cell lines show a 45% reduction in growth rate from the parental MCF7, and the intermediate HER2 expressing clones exhibit an 85% reduction in growth rate (*P*<0.05). The subclones in the high HER2 overexpressing group exhibit higher growth rates than the subclones with intermediate levels of HER2 expression. These findings do not support a monotonic relationship between mean HER2 expression level and growth rate.

### Cell by cell correlation of HER2, EGFR and HER3 expression levels in the HER2 transfectants

**Figure 2:**
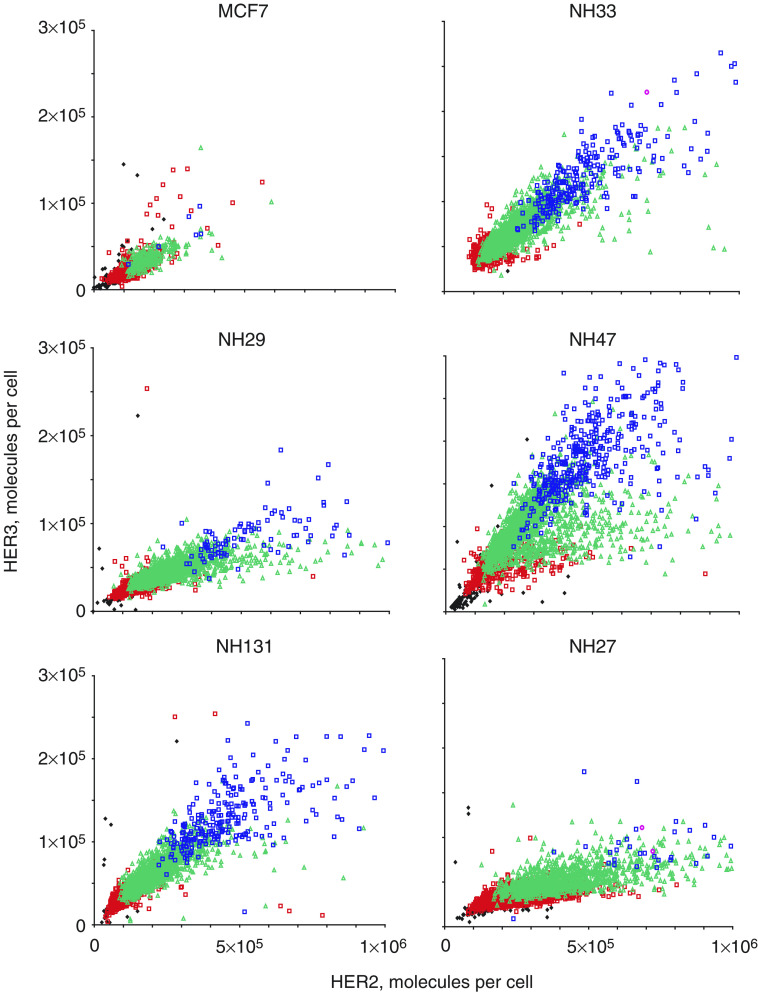
Laser scanning cytometry analysis of EGFR, HER2 and HER3 expression in parental MCF7 and MCF7 HER2 transfected subclones. Parental MCF7 and HER2 transfected subclones were trypsinised, stained with fluorochrome-conjugated antibodies directed against the EGFR, HER2 and HER3, and subjected to multiparameter LSC analysis. Correlated cell-by-cell fluorescence measurements are shown with HER2 expression plotted on the abscissa, HER3 expression plotted on the ordinate, and EGFR expression plotted as object/colour coded: EGFR <20 000 molecules cell^−1^ (black diamonds), EGFR 20 000 to <40 000 molecules per cell (open red squares), EGFR 40 000 to <80 000 molecules per cell (open green triangles), EGFR>80 000 molecules per cell (open blue squares). All receptor level values are expressed as receptor numbers per cell, based on known mean values (determined independently by ELISA) in a concomitantly run reference cell line, and corrected for autofluorescence, cell aggregates and fluorescence channel crosstalk.

Parental MCF7 cells and HER2 transfected MCF7 subclones were grown to subconfluence, and fed with normal culture media 24 h prior to trypsinisation and cell fixation. The fixed cells were stained with fluorochrome conjugated antibodies against the EGFR, HER2 and HER3 and analyzed by LSC. The results, expressed in molecules per cell and correlated cell by cell, are shown in Figure 2. The HER2 level for each cell is plotted on the abscissa, and the HER3 level for each cell is plotted on the ordinate. The EGFR level for each cell line is shown using a series of colour-coded symbols as indicated in the figure legend. In all cell lines, there is a correlation between levels of HER2/cell and levels HER3/cell in the same cells, as well as between HER2/cell and EGFR/cell, and EGFR/cell and HER3/cell (see Supplementary Table 1S).

It is apparent that despite the overall correlations among levels of HER2, EGFR and HER3 in the same cells, there are substantial differences among the different subclones with respect to the ranges of levels of expression of these cell constituents. Human epidermal growth factor receptor 2 levels are lowest in the parental MCF7 cell line and highest in NH27 cells, but NH27 cells have neither high levels of HER3 nor the highest levels of EGFR per cell. In most of the MCF7 subclones that contain high EGFR overexpressing cells, the cells with EGFR levels exceeding 80 000 molecules per cell exhibit only moderately elevated levels of HER2 (mostly in the range of 200 000–600 000 molecules per cell). However, in NH27 cells, among the few that exhibit EGFR levels in excess of 80 000 molecules per cell, most also have HER2 levels that exceed 600 000 molecules per cell. Thus, across subclones, there is a relationship between levels of EGFR expression and HER3 expression per cell that is worth pursuing (see below), but there does not appear to be a strict quantitative relationship between increased EGFR levels and/or HER3 levels on the one hand and increasing HER2 levels on the other.

### Cell by cell levels of P-MAPK and P-AKT

**Figure 3:**
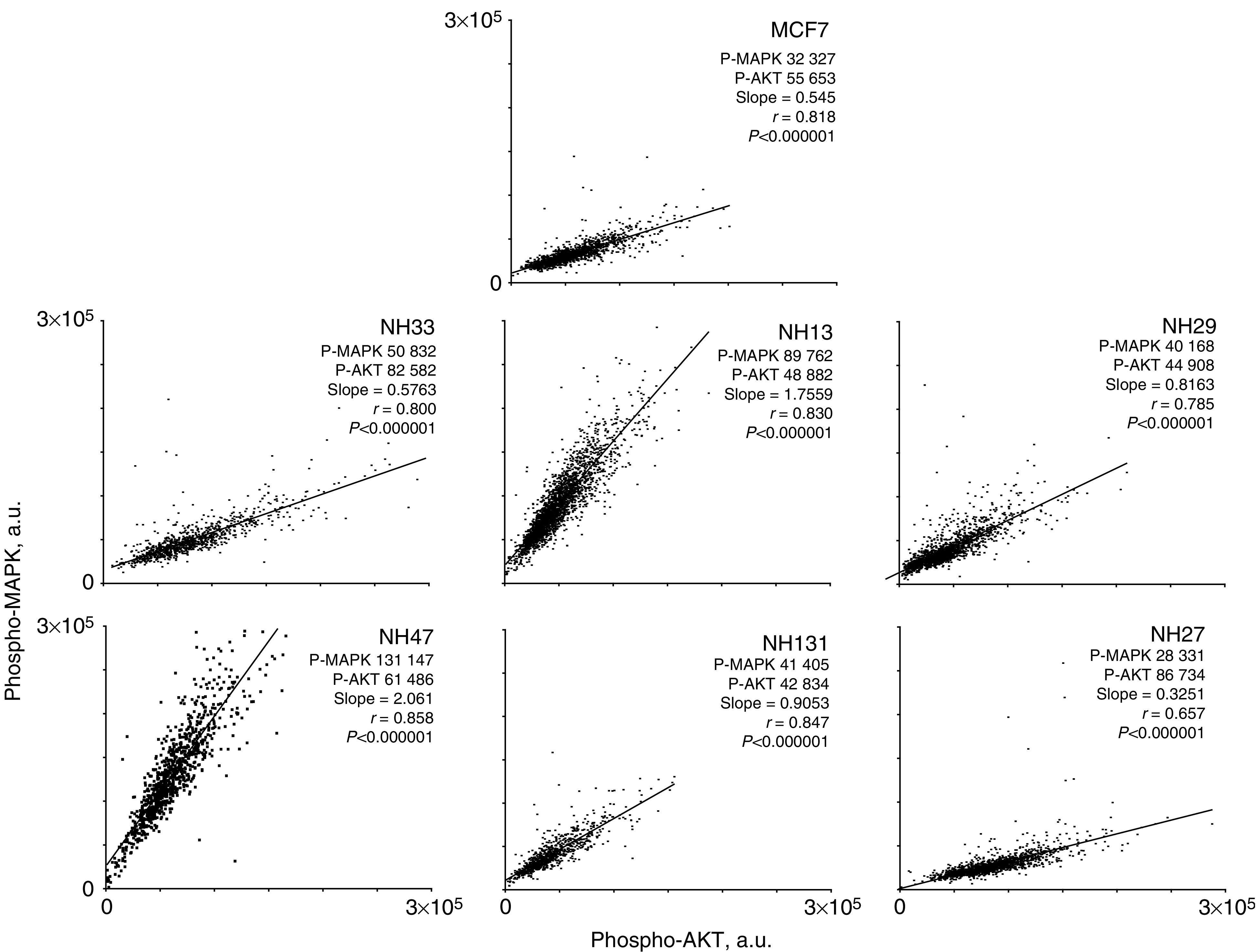
Laser scanning cytometry analysis of P-MAPK and P-AKT levels on a cell-by-cell basis in control and HER2 transfected MCF7 subclones. For each cell, P-MAPK level per cell is plotted on the ordinate and P-AKT level in the same cell is plotted on the abscissa. Values are corrected for autofluorescence, cell aggregates and cross talk. The mean fluorescence values for P-MAPK and P-AKT, the slope, and correlation coefficient (*r*) for each cell line are shown.

Parental MCF7 cells and HER2 transfected MCF7 subclones were prepared for LSC, and stained with antibodies against the phosphorylated forms of MAPK and AKT. Correlated cellular levels of P-MAPK and P-AKT were determined by LSC (Figure 3). Quantitative cellular levels of each phospho-protein are expressed in arbitrary units, where mean cellular levels obtained in concomitantly run aliquots of stored reference cells are assigned values of 10 000 arbitrary units per cell. Mean levels of P-AKT range from ∼43 000 units per cell in NH131 cells to ∼87 000 units per cell in NH27 cells. Across cell lines, there is no strong correlation between mean P-AKT level per cell and HER2 level (*r*=0.078, *P*=0.868; see Supplementary Figure 1S).

There was considerable variation in levels of P-MAPK per cell, both within and among the different cell lines (Figure 3). Across cell lines, mean levels of P-MAPK per cell varied from ∼28 000 units per cell in NH27 cells to ∼131 000 units per cell in NH47 cells. There was no correlation between mean P-MAPK level per cell and HER2 level across cell lines (*r*=−0.067, *P*=0.886; see Supplementary Figure 1S).

Within individual cell lines, the simultaneous measurement of P-MAPK and P-AKT in each cell by LSC reveals a strong correlation between the cellular levels of these two phospho-proteins (Figure 3). However, the slopes of the regression vary from cell line to cell line.

### Effects of AG1478 and trastuzumab on cell proliferation

**Figure 4:**
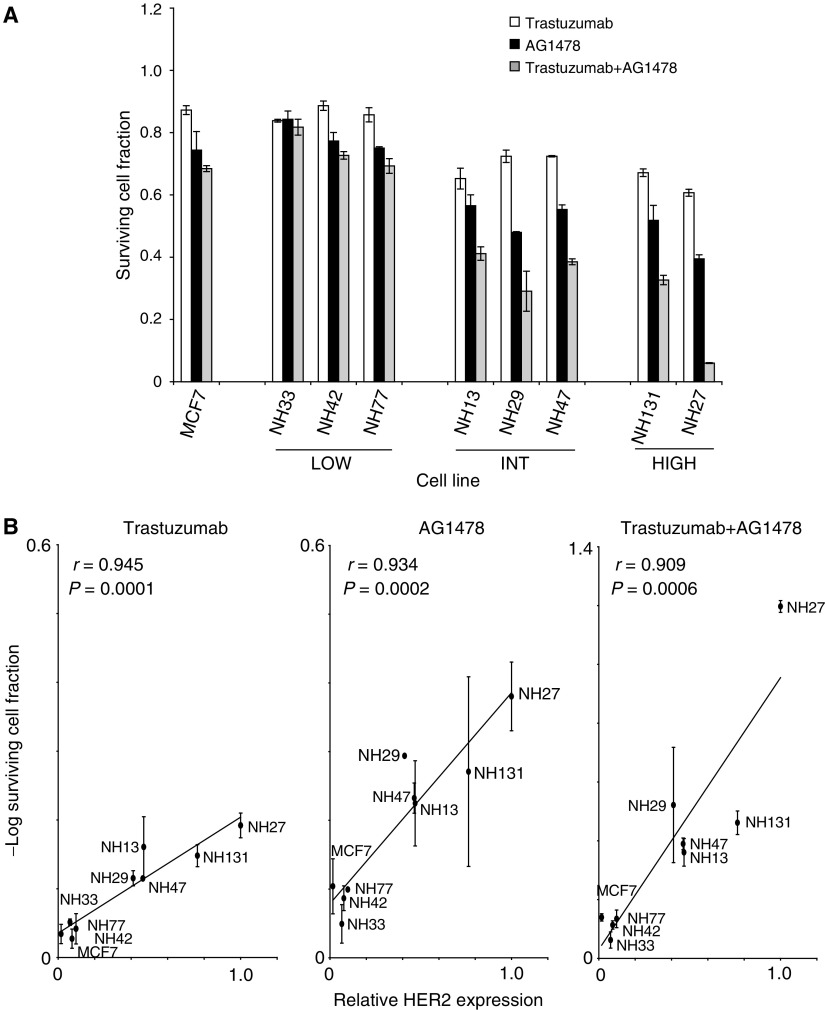
Effects of AG1478, trastuzumab, and a combination of AG1478 and trastuzumab on the growth of control cell lines and HER2 transfected subclones, and correlation of HER2 expression with drug efficacy. (**A**) Parental MCF7 control cell lines and the HER2 transfected MCF7 subclones were plated into 12-well tissue culture plates and treated with 1 *μ*g ml^−1^ trastuzumab (white bars), 500 nM AG1478 (black bars), or a combination of both (grey bars) for 5 days. On day 6, the cells were trypsinised, counted and the cell number from drug treated samples were normalised with respect to control cells treated with vehicle only (DMSO and human IgG). (**B**) Mean HER2 expression level in the cell lines, normalised with respect to the HER2 content of the NH27 cell line, was correlated to –log of surviving cell fractions from individual drug treatments (Trastuzumab, and AG1478) and combined drug treatments (Trastuzumab+AG1478). −log of surviving cell fraction is plotted on the ordinate, and mean HER2 expression is plotted on the abscissa. The correlation coefficient (*r*) and *P*-value for each correlation are shown. The results are from at least three independent experiments. Bars=±95% CI.

The effects of 0.5 *μ*M AG1478, 1 *μ*g ml^−1^ trastuzumab, and a combination of 0.5 *μ*M AG1478 and 1 *μ*g ml^−1^ trastuzumab on cell proliferation are shown in Figure 4A. These drug concentrations were estimated to fall within a clinically achievable range (Hidalgo *et al*, 2001; Pegram *et al*, 2004), and showed activity in preliminary dose response studies (see Supplementary Figure 2S). In transfected MCF7 cell subclones with modest increases in HER2 overexpression (NH33, NH42 and NH77), the effects of trastuzumab on cell proliferation (white bars, Figure 4A) are not significantly different from its effects on parental MCF7 cells. Trastuzumab decreases cell proliferation in the intermediate HER2 overexpressing group by an average of 17% in comparison with parental MCF7 cells (*P*=0.005). In the high HER2 expressing group, trastuzumab produced an average decrease in cell proliferation that is 24% greater than in parental MCF7 cells (*P*=0.004), but the difference in the effects of trastuzumab on intermediate and high HER2 overexpressing MCF7 subclones is not statistically significant (*P*=0.244).

AG1478 treatment does not substantially inhibit the growth of parental MCF7 or the low HER2 overexpressing subclones (black bars, Figure 4A). Compared to parental MCF7, AG1478 has a 25 and 36% greater mean inhibition of proliferation on the intermediate (*P*=0.05) and high (*P*=0.007) HER2 expressing clones, respectively. However, the difference in response between the intermediate and high HER2 expressing clones is not statistically significant (*P*=0.077).

Combined treatment of these cell lines with both AG1478 and trastuzumab (grey bars, Figure 4A) produces a significantly greater inhibition of proliferation than that of either agent alone only in cell lines with intermediate and high levels of overexpression of HER2. The drug combination produces greater inhibition of cell proliferation in the intermediate and high HER2 expressing clones in comparison with AG1478, the best single agent for these cell lines (*P*<0.05).

When all of the cell lines in the panel are considered together, there are strong correlations between log growth inhibition by each drug individually or in combination and mean levels of HER2 expression in the cell lines (Figure 4B). There is a strong correlation between the degree of cell growth inhibition by trastuzumab and HER2 expression level (*r*=0.945, *P*=0.0001; Figure 4B, left panel), between AG1478 and HER2 expression level (*r*=0.934; *P*=0.0002; Figure 4B, middle panel), and between the degree of cell growth inhibition by combined treatment (AG1478 plus trastuzumab) and HER2 expression level (*r*=0.909, *P*=0.0006; Figure 4B, right panel).

To further characterise the interaction between the effects of AG1478 and trastuzumab, the CI was calculated for each cell line (Table 1). Overall, the CIs suggest that the combined drug effect is additive, except in the NH27 cell line, where synergy was clearly demonstrated. However, since the overall effects of these agents are small in the low HER2 expressing lines, the CI results may be difficult to interpret in these cell lines.

### Prediction of efficacy of AG1478, trastuzumab and the combination of AG1478 and trastuzumab from multiple receptor expression data

We applied least-squares regression to find a best-fit linear model of drug efficacy as a function of mean EGFR, HER2 and HER3 expression levels derived from LSC measurements of parental MCF7 and HER2 transfected subclones (Table 2). Using the three receptor levels individually as predictors of trastuzumab efficacy with leave-one-out cross-validation, the root mean square prediction errors are 0.099 for HER2 alone, 0.123 for EGFR alone, and 0.159 for HER3 alone. Thus, HER2 is the best individual marker for the prediction of trastuzumab efficacy in these cell lines. With regard to predicting for AG1478 effect, root mean square errors are 0.192 for HER2 alone, 0.233 for EGFR alone, and 0.286 for HER3 alone, suggesting that HER2 expression level is also the best single predictor of AG1478 efficacy in these cell lines. Human epidermal growth factor receptor 2 is also the best individual predictor for combined drug efficacy.

Overall, the smallest root mean square error for prediction of trastuzumab efficacy (0.089) was achieved using a combination of HER2 and HER3 expression levels. Interestingly, the overall smallest root mean square error for prediction of AG1478 efficacy (0.180) is achieved using a combination of EGFR and HER3 expression levels. Predictions using all three receptor expression levels together result in errors that were not as low as those derived from the best individual or two-receptor predictions, suggesting that the data set may be too small to draw robust inferences from the three-protein model.

## DISCUSSION

It is generally agreed that there is a need for better clinical biomarkers for response to EGFR targeted therapy (Baselga, 2002; Castro, 2002; Arteaga and Baselga, 2003). Overall clinical response rates to EGFR targeted therapy in many of the common human solid tumours are generally low, often in the range of 5–20% (Castro, 2002; Fukuoka *et al*, 2003; Kris *et al*, 2003). Many patients whose tumours express EGFR fail to respond to EGFR targeted therapy, and conversely, patients who respond may have tumours that do not exhibit EGFR expression (Noberasco *et al*, 2005; Younes, 2005). Preclinical studies in tissue culture and in xenografts using cancer cell lines with known EGFR expression have shown that EGFR overexpression itself might not be a useful predictive marker for response to EGFR targeted therapy (Sirotnak *et al*, 2000; Ciardiello *et al*, 2000, 2001; Moasser *et al*, 2001; Wakeling *et al*, 2002; Campiglio *et al*, 2004). In this study, we show that growth inhibition in response to AG1478, a targeted inhibitor of the EGFR, and AG1478 in combination with the HER2 targeted antibody trastuzumab, is correlated with increased HER2 expression levels. The largest effects of these therapies are seen in the cell lines expressing the highest levels of HER2.

Human epidermal growth factor receptor 2 is known to heterodimerise preferentially with the EGFR (Graus-Porta *et al*, 1997), potentiate mitogenic signalling by increasing ligand affinities of the EGFR, and protect the EGFR from degradation (Tzahar *et al*, 1996; Lenferink *et al*, 1998). This could provide a mechanistic explanation for our findings. In support of this premise, EGFR and HER2 co-expression has been linked to a more aggressive clinical phenotype and to poor prognosis in breast cancer (Thor *et al*, 2001; Suo *et al*, 2002). Preclinical studies in breast cancer cell lines that overexpress HER2 show that combined EGFR and HER2 inhibition may be more effective than treatment with single agents, regardless of EGFR expression level (Moasser *et al*, 2001; Moulder *et al*, 2001; Normanno *et al*, 2002; Anido *et al*, 2003).

Our panel of HER2-transfected subclones was intended to provide the opportunity to study the effects of targeted therapeutic agents in a family of cell lines that have few overall genotypic differences, and that express graded levels of HER2 protein. Nonetheless, there are differences among these subclones in quantitative levels of intracellular co-expression of key proteins, including HER2, EGFR and HER3 (Figure 2), and relative levels of co-expression of P-ERK and P-AKT per cell, which also vary from cell line to cell line (Figure 3). Thus, we demonstrate a direct relationship between the degree of efficacy of anti-EGFR regimens and the levels of HER2 expression in breast cancer cell lines (Figure 4A and B). This supports the case for investigating the potential clinical role of HER2 overexpression as a predictive marker for response to anti-EGFR therapy in human cancers.

Other cell constituents might also predict response to anti-EGFR therapy, and some might provide independent predictive information. The data on EGFR, HER2 and HER3 expression levels by LSC support this possibility. We have noted that high cellular levels of EGF receptor are often accompanied by high levels of HER3 in the same cells (Figure 2). The linear regression analysis shows that although increased expression of HER2 is the best single predictor for responsiveness to AG1478 or the AG1478-trastuzumab combination (as well as for trastuzumab alone), the best *combination* of predictors for the efficacy of AG1478 or combined AG1478-trastuzumab is increased expression of the EGFR and HER3 (Table 2).

Of course, one cannot assume that results obtained on cell lines *in vitro* can be translated directly into the clinic. Additional support from experimental studies *in vivo* would be highly desirable. Such studies would require the development and validation of a suitable panel of tumour lines that stably overexpress graded levels of HER2 *in vivo*.

Several published clinical studies suggest that our findings in breast cancer cell lines might be of some relevance in the clinical setting. In a recent study of breast cancer patients, 35% of 306 HER2-overexpressing tumours were found to express EGFR Conversely, 87% of EGFR-overexpressing tumours were found to overexpress HER2 as well (DiGiovanna *et al*, 2005). In a recent paper involving NSCLC patients, clinical responses to gefitinib (an anti-EGF receptor agent) were shown to be correlated with HER2 overexpression/amplification in EGF receptor-positive patients (Cappuzzo *et al*, 2005). It would seem reasonable to investigate the possibility of a relationship between HER2 status and response to anti-EGFR therapy in patients with breast cancer as well, based on our findings in breast cancer cell lines that responses to trastuzumab and AG1478 alone or in combination are correlated with high levels of HER2 expression.

In summary, we show that in human breast cancer cell lines increased levels of HER2 expression alone are associated with increased effectiveness of anti-EGFR therapy, alone or in combination with anti-HER2 therapy, and that the combination of EGFR and HER3 overexpression may be an even better predictor of response. This would suggest the possibility that HER2 overexpression alone and/or the combination of EGFR and HER3 expression levels might be useful clinical markers for response to EGFR and combined EGFR–HER2 targeted therapy in patients with breast cancer.

## Figures and Tables

**Table 1:** Mean survival fractions and combination indices for trastuzumab treatment, AG1478 treatment, and AG1478+trastuzumab combination treatments

	**Mean surviving fraction with AG1478**	**Mean surviving fraction with trastuzumab**	**Mean expected survival fraction for additivity**	**Mean observed survival fraction with the AG1478+trastuzumab combination**	**Mean combination index**	***P-*value (combination index)**
MCF7	0.788±0.037	0.924±0.009	0.727±0.027	0.725±0.006	0.999±0.046	0.998
NH33	0.893±0.028	0.888±0.005	0.793±0.028	0.866±0.016	1.093±0.018	0.034
NH42	0.866±0.050	0.939±0.017	0.813±0.051	0.770±0.008	0.955±0.065	0.561
NH77	0.784±0.012	0.929±0.022	0.728±0.019	0.734±0.014	0.985±0.030	0.671
NH13	0.599±0.043	0.691±0.021	0.415±0.038	0.436±0.014	1.064±0.076	0.486
NH29	0.508±0.001	0.767±0.012	0.390±0.005	0.308±0.040	0.788 ±0.091	0.145
NH47	0.586±0.016	0.767±0.002	0.450±0.014	0.408±0.010	0.909±0.040	0.149
NH131	0.549±0.082	0.711±0.008	0.391±0.060	0.346±0.009	0.932±0.154	0.701
NH27	0.418±0.025	0.630±0.004	0.264±0.017	0.064±0.001	0.243±0.014	0.0003

Mean survival fraction and combination index (CI) values were obtained from triplicate studies. The *P*-values for each cell line indicate the level of statistical significance of the CI compared with a CI value of 1.0. Mean CI values significantly greater than 1.0 (*P*<0.05) indicate antagonism, and values significantly less than 1.0 (*P*<0.05) indicate synergy. Numbers in parentheses=standard error (s.e.). CI=combination index.

**Table 2:** Comparison of root mean square errors of predicted AG1478 and trastuzumab treatment outcomes from receptor expression levels

	**HER2**	**EGFR**	**HER3**	**EGFR+HER2**	**EGFR+HER3**	**HER2+HER3**	**All**
Trastuzumab	0.099	0.123	0.159	0.159	0.133	0.089	0.146
AG1478	0.192	0.233	0.286	0.344	0.180	0.286	0.284
Combination	0.303	0.370	0.442	0.509	0.255	0.442	0.402

Least-squares linear regression analysis was applied to receptor expression data from LSC analysis and inhibitor proliferation assay results to determine the correlation between receptor expression levels and drug treatment outcomes as described in the Materials and Methods. Predictions were made from individual receptor expression, two receptor combinations, and all three receptors together on each of the drug combinations tested. Shown is the root mean square errors derived from each drug–receptor combination.
