# Supplementary figures and images for: HER2 expression as a potential marker for response to therapy targeted to the EGFR
Source: Br J Cancer. 2006 Apr 4;94(8):1144–53. doi: 10.1038/sj.bjc.6603078 (PMC2361260; doi:10.1038/sj.bjc.6603078)

Figure 1 supplement

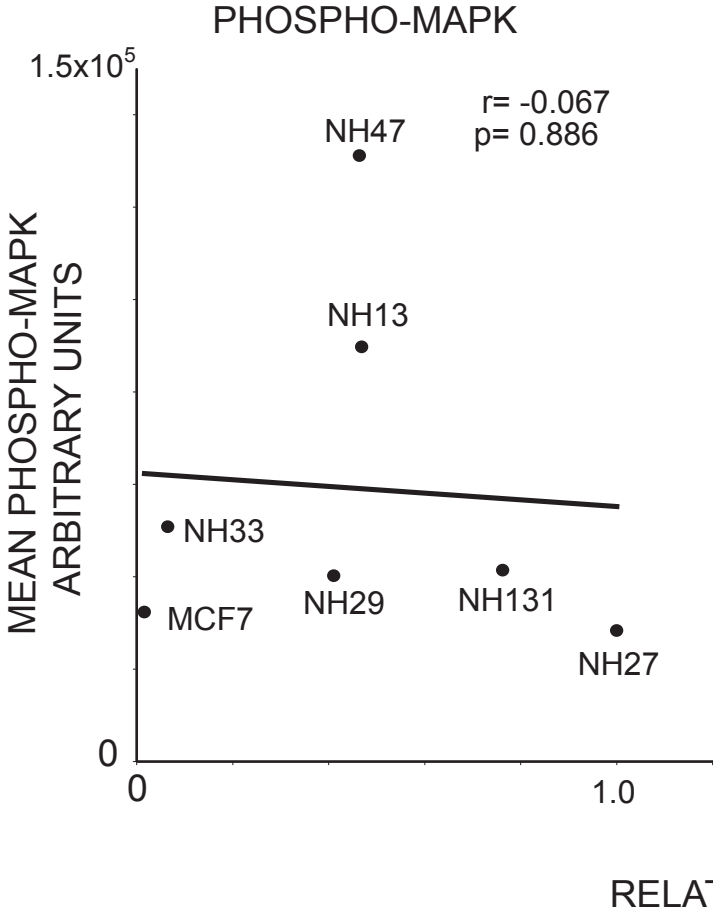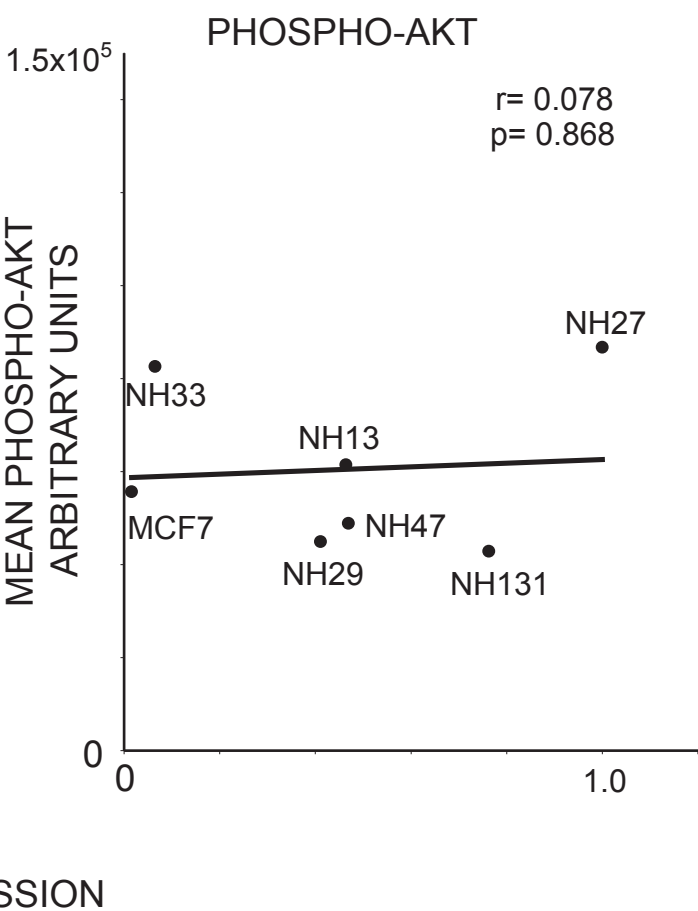

Supplement: Supplementary Figure S1 [file 94-6603078x1.pdf]

A

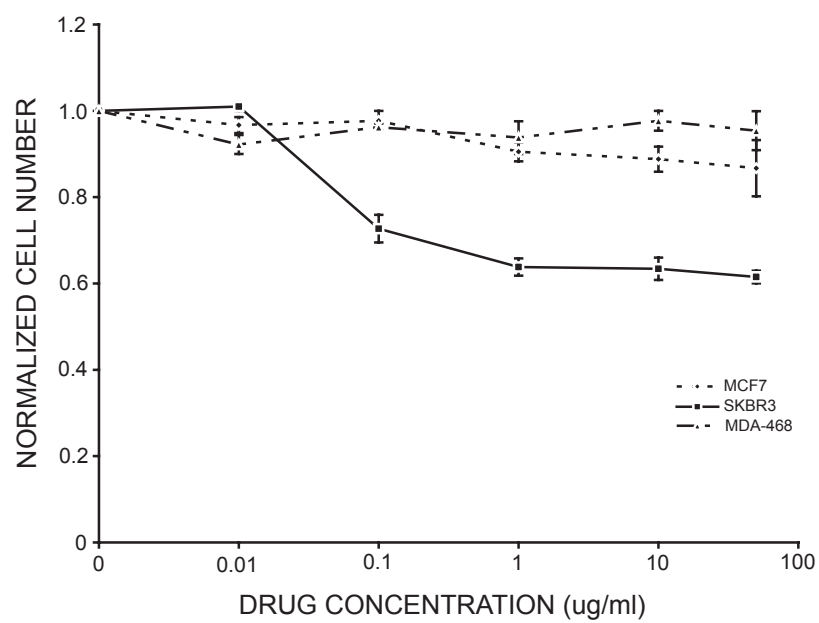

B

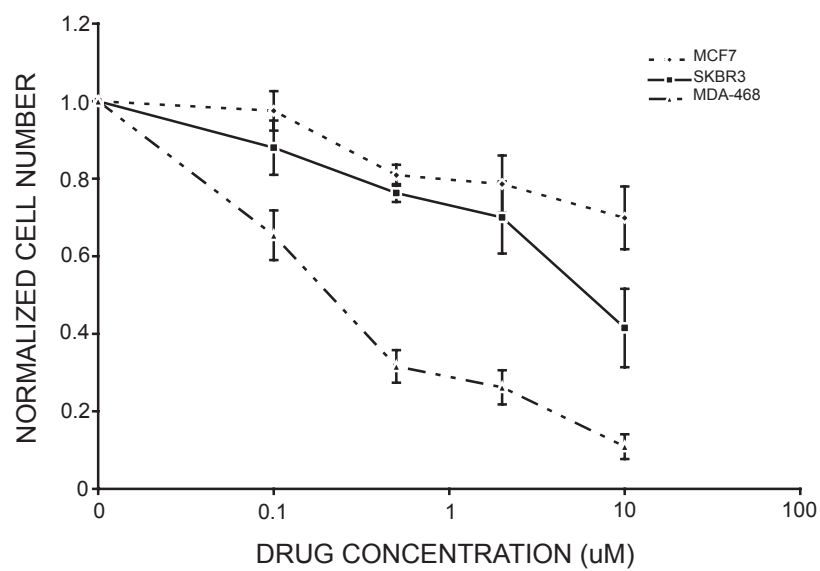

Supplement: Supplementary Figure S2 [file 94-6603078x2.pdf]

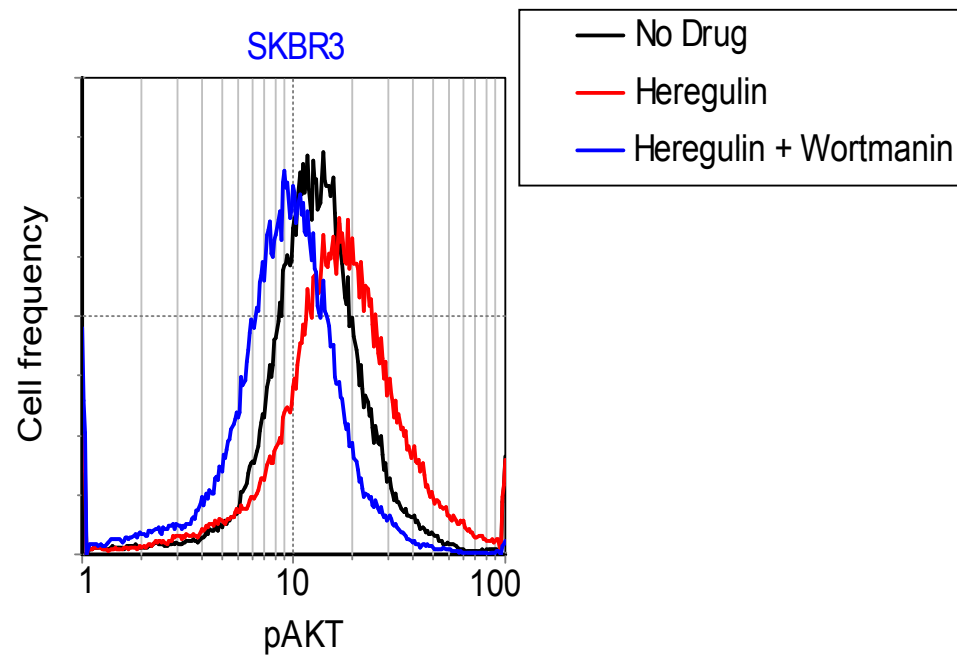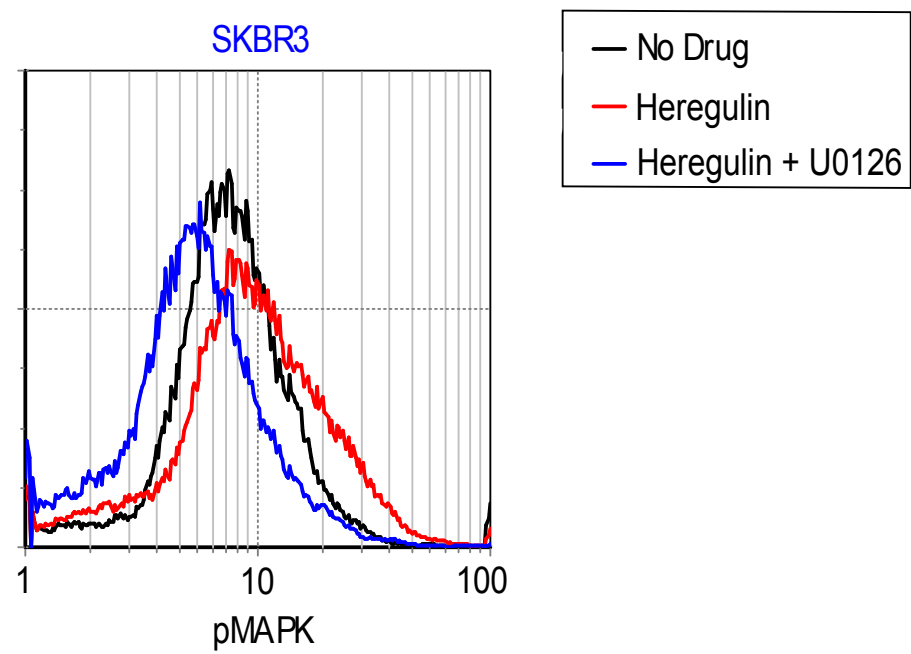

Supplement: Supplementary Figure S3 [file 94-6603078x3.pdf]

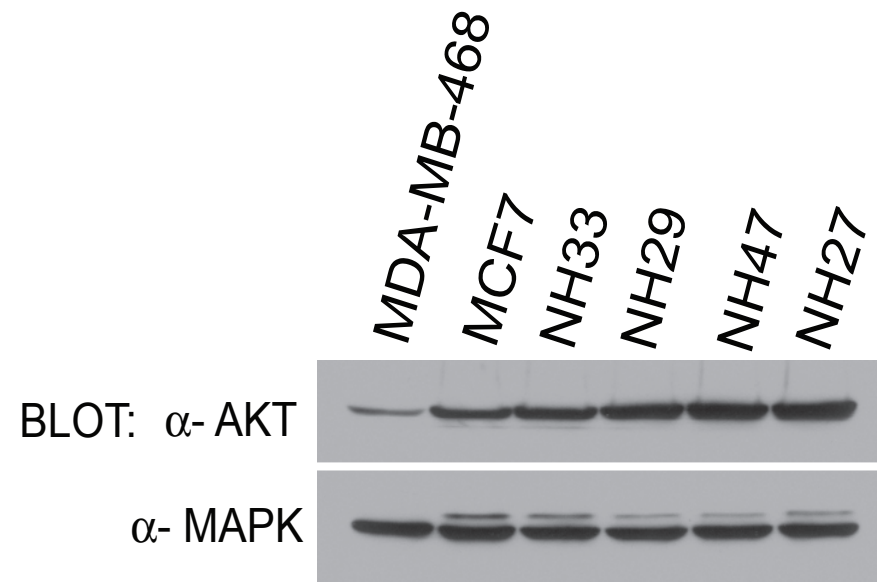

Supplement: Supplementary Figure S4 [file 94-6603078x4.pdf]
