# Supplementary material for: HER2 expression as a potential marker for response to therapy targeted to the EGFR
Source: Br J Cancer. 2006 Apr 4;94(8):1144–53. doi: 10.1038/sj.bjc.6603078 (PMC2361260; doi:10.1038/sj.bjc.6603078)
Supplement: Supplementary Table S1 [file 94-6603078x5.pdf]

Table 1-S.

Cell Line	HER2-EGFR	HER2-HER3	EGFR-HER3
SKBR3	0.71	0.739	0.795
MCF7	0.687	0.725	0.552
NH33	0.745	0.832	0.814
NH29	0.798	0.779	0.836
NH47	0.657	0.668	0.910
NH131	0.847	0.855	0.895
NH27	0.763	0.773	0.857
