# Supplementary material for: HER2 expression as a potential marker for response to therapy targeted to the EGFR
Source: Br J Cancer. 2006 Apr 4;94(8):1144–53. doi: 10.1038/sj.bjc.6603078 (PMC2361260; doi:10.1038/sj.bjc.6603078)
Supplement: Supplementary Figure and Data Table Legends [file 94-6603078x6.doc]

Supplementary Figure and Data Table Legends

*Table 1-S. Correlations among cell-by-cell levels EGFR family member expression in control cell lines and HER2 transfected sub-clones.* The correlation coefficient for each receptor pair in each cell line analyzed is shown. The correlation coefficient for the SKBR3 cell line was included for comparison to the MCF7 panel. P values are all < 1x10^-6^, except for the correlation between HER2 and HER3 expression in NH131 cells, where P = 0.009.

*Figure 1-S.. Correlation of Phospho-MAPK and Phospho-AKT levels with HER2 Expression in parental MCF7 Cells and the HER2 Transfected MCF7 Sub-clones.* Mean HER2 expression level in the cell lines, normalized with respect to the HER2 content of the NH27 cell line, was correlated to mean P-MAPK and P-AKT levels derived from the LSC analysis in figure 3. Mean P-MAPK or P-AKT levels are plotted in arbitrary units on the ordinate, and HER2 expression is plotted on the abcissa. The correlation coefficient (r) and *P* value (p) for each correlation are shown.

*Figure 2-S Dose response analysis of AG1478 and trastuzumab on control cell lines.* SKBR3 and Parental MCF7 control cell lines were seeded into 12 well tissue culture plates, and fed and treated with increasing concentrations of a) trastuzumab or b) AG1478 every other day for 5 days. On day 6, the cells were trypsinized, counted and cell number was normalized to samples treated with vehicle only (DMSO or human IgG). Error bars represent the 95% confidence interval from the average of three independent experiments. Responses of MDA-MB-468 cells shown for reference.

*Figure 3-S .Flow cytometry analysis of P-MAPK and P-AKT levels in response to cell stimulation or inhibition.*

*Figure 4-S. Immunoblots of total MAPK and ERK in the MCF7 parental line and the HER2 overexpressing sublines. Levels in MDA-MB-468 cells, an unrelated non-HER2-overexpressing cell line shown for reference.*
